# Obituary—Dr. John Allen

**Published:** 1892-05

**Authors:** 


### Obituary—Dr. John Allen.

Died at his home in Plainville, N. J., March 8, 1892, Dr. John Allen, D.D.S. the oldest practicing dentist in New York City.

The Doctor's death was occasioned by physical exhaustion.

A brief biographical sketch may here be made.

He was born in Broom County, New York, November 4, 1810. He was a descendant of the Ethan Allen family of Vermont, whose history is identified with the Revolutionary War. His father, Dr. N. Allen, moved to Ohio when John was but a boy. Here agriculture engrossed the attention of the family for several years. At the age of nineteen this son became a student of Dr. James Harris, a medical man of high standing, who relinquished the practice of medicine for that of dental surgery. After having concluded his course of pupilage with Dr. Harris he began his professional career in Cincinnati, in 1830; here in the early part of his practice, with time on his hands, he availed himself of the advantages of the Ohio Medical College, of Cincinnati, for acquiring a more thorough foundation for the practice of this specialty. Being the only dentist in the college it was agreed by the students of the class that when subjects were obtained for dissection he should have the teeth for experiment and use in his practice—not only human teeth but those of cows and other animals were also obtained and utilized in the preparation of substitutes for the natural teeth. Dentures were also carved from the teeth of the hippopotamus and used for the purpose, porcelain teeth not having as yet been brought into use. Soon after this, however, these were made and generally used. Dr. Allen sought and obtained a practical knowledge of their manufacture. Single and block teeth, when well mounted upon gold plates, were considered the highest style of artificial dentistry that had then been obtained. There were, however, still, defects that he sought to overcome; and although he had reached the maximum in this style of work, he sought to attain more perfect results by which he might avoid fissures, the stiff mechanical appearance, and in many instances, a failure to restore the proper form and expression of the mouth and face. To meet this apparent demand for some mode by which more perfect results could be obtained, Dr. Allen resolved to commence various experiments with a view of working out a new system, which he had conceived, which was yet vague and chaotic—a mere germ. But the method of developing the system led through a dark way, along an untrodden path, with little or no antecedent light. He persevered, however, and his efforts were crowned with success. He did succeed in restoring lost parts in such a manner as to bring back the original features of the face in a very marked degree; he succeeded not only in doing this but in producing the most perfect dentures that had ever been devised, especially in reference to the absolute closure of all interstices, the complete arrangement of teeth to correspond with the features of the face to secure good enunciation, and the accomplishment of thorough mastication. His success was fully recognized by the dental profession in both this and European countries. Dr. Allen's improvements were taken up and adopted in practice by many of the leading dentists in this country, and subsequently in other countries. The well-constructed dentures of continuous gum work are acknowledged upon all hands to be much superior to any other style of dentures. This much is here said because the perfecting of this work was the great achievement of Dr. Allen's life, and his name will be associated with it so long as artificial dentures are needed and constructed.

Notwithstanding Dr. Allen's time and efforts were so largely bestowed in the particular line of work here indicated, he had time and effort for the general welfare of his chosen profession. He was always interested in educational matters—himself at one time being a college professor. He was during all the active years of his life connected with and took part in association work. He was a good writer, and contributed to the literature of the profession. He was a man beloved by all who knew him, and highly appreciated and respected by all who knew of him, and it may truthfully be said of him he was a high type of a Christian gentleman.

A more extended sketch of Dr. Allen was published in the April No. of Vol. 28 of the Dental Register.

